# *Acta Oncologica* Nordic Precision Cancer Medicine Symposium 2023 – merging clinical research and standard healthcare

**DOI:** 10.2340/1651-226X.2024.24954

**Published:** 2024-06-23

**Authors:** Elisa Bjørgo, Gro L. Fagereng, Hege G. Russnes, Sigbjørn Smeland, Kjetil Taskén, Åslaug Helland

**Affiliations:** a MATRIX, Norwegian Centre for Clinical Cancer Research, Division of Cancer Medicine, Oslo University Hospital, Oslo, Norway; b Institute for Cancer Research, Division of Cancer Medicine, Oslo University Hospital, Oslo, Norway; c Institute of Clinical Medicine, University of Oslo, Oslo, Norway; d Department of Pathology, Oslo University Hospital, Oslo, Norway; e Division of Cancer Medicine, Oslo University Hospital, Oslo, Norway

**Keywords:** Precision cancer medicine, molecular diagnostics, biomarkers, clinical trials, DRUP-like clinical trials, health economics

## Introduction

*Acta Oncologica* has for several decades supported Nordic cancer-related symposia, and in 2023 a new biannual *Acta Oncologica* Nordic Precision Cancer Medicine Symposium (NPCM) series was initiated. The first NPCM conference ‘Merging Clinical Research and Standard Healthcare’ took place in Oslo, September 17–19 2023 and was hosted by Oslo University Hospital and the Norwegian Centre for Clinical Cancer Research, MATRIX. Over 2 days, the conference gathered participants from key precision medicine environments from Australia, the US, and Europe.

Precision cancer medicine is changing oncology through advanced molecular profiling, innovative clinical trials, and an increasing number of targeted drugs and treatment options. Identified molecular properties may explain why patients with the same type and stage of cancer respond differently to the same treatment. For the precision cancer medicine approach to have an impact and move towards implementation in national healthcare systems, it is essential to have access to both advanced molecular diagnostics and drugs. Although the promise of precision cancer medicine is clear and novel anti-cancer drugs targeting genetic alterations enter the market every year, implementation is still challenging. Access to these approaches is unequal due to varying availability of adequate molecular diagnostics, uncertainties regarding real-world effectiveness, hurdles regarding co-payment and reimbursement, and limited access to clinical trials and early access programmes.

Over the last decade, several national initiatives have addressed the challenges with implementation of precision cancer medicine, and during the NPCM 2023 conference, the different initiatives gathered to share and discuss key learnings and synergy potential of international collaboration within this field.

The first Nordic Precision Cancer Medicine Symposium brought together experts from different areas important for precision cancer medicine implementation into standard healthcare, and topics addressed during the conference included molecular pathology and molecular tumour boards (MTBs), biomarkers for stratification, clinical study design, DRUP-like clinical trials, scaling of precision medicine ecosystems as well as health economics, implementation, and policies. In this special edition focusing on precision medicine, altogether 10 speakers and poster presenters at the NPCM publish recent precision cancer medicine updates.

## Keynote lectures: Precision cancer medicine from bench to bed

Three keynote speakers presented new developments within the precision cancer medicine field at the NPCM 2023, including presentations on cutting-edge molecular diagnostics, the Australian implementation initiative, and regulatory developments.

Gordon Mills from the Knight Cancer Institute, Oregon Health & Science University, presented a new clinical study design, targeting adaptive responses in cancer. Malignant cells and the tumour environment adapt to therapy. In the Serial Measurements of Molecular and Architectural Responses to Therapy (SMMART) trial, the patient’s cancer is followed over time through serial biopsies and comprehensive analysis of tumour cells and the tumour ecosystem. Drug and drug combinations are subsequently adjusted based on these analyses to avoid resistance. A big challenge of multi-drug treatment is to measure adaptive responses in real-time, and tools beyond RECIST criteria are therefore required.

David Thomas from the Garvan Institute of Medical Research in Sydney gave an overview of the Australian precision cancer medicine initiatives. Omico has established comprehensive genomic profiling for patients with advanced or incurable cancer. The national Molecular Screening and Therapeutics study enrols patients with incurable cancer and has so far recruited 750 patients, and the new PrOSPeCT programme is a precision oncology screening platform enabling clinical trials by linking genomic technology to trials of new therapeutic products. Thus, Australian patients with advanced cancer have access to systematic precision cancer medicine.

Francesco Pignatti from the European Medicines Agency presented pan-cancer drug development from a regulatory perspective. Pignatti addressed some of the challenges with tumour-independent indications and approving drugs based on single-armed trials. Pignatti concluded that approval of biomarker-driven indications is similar to other approvals in high-unmet need situations. Moreover, the importance of addressing knowledge gaps prior to an approval process was emphasized.

## Conference Sessions: Sharing experiences and highlighting collaboration for implementation of precision cancer medicine

The NPCM conference consisted of five conference sessions addressing molecular precision diagnostics and MTBs, design of clinical trials, health economics, implementation and guidelines, scaling of precision medicine ecosystems, and the growing ecosystem of DRUP-like clinical trials. In each session, three internationally invited speakers presented front-line research connected to the topic. In addition, short talks selected from abstract submissions were included.

Session one, Molecular pathology and MTBs, addressed advanced precision diagnostics. Access to adequate molecular profiling is crucial for the success of precision medicine. The three invited speakers in this session, Funda Meric-Bernstam from the MD Anderson Cancer Centre in Houston, Texas, Maud Kamal from Institut Curie in Paris, and Lynette Sholl from the Bringham and Women’s Hospital and Harvard Medical School in Boston, highlighted key learnings from ongoing initiatives. Meric-Bernstam emphasized that a comprehensive analysis on DNA/RNA/protein is necessary to improve patient selection and treatment planning. Kamal gave an overview from their MTB and highlighted the need for clinical practice guidelines in genomic testing as well as the need to provide decision support tools and train physicians to interpret genomic data. Sholl gave an overview of the institutional cancer profiling in Boston, where more than 45,000 patients have already been screened. She addressed that 10–20% of cancer patients harbour a germline alteration conferring cancer susceptibility, and that testing for tumour-only misses important germline variants. A paired tumour-germline testing platform has therefore been established and implemented in Boston. Sholl emphasized that operationalising routine germline testing for cancer patients requires substantial inter-disciplinary teamwork. In this *Acta Oncologica* special edition, two NPCM short talk speakers present new findings highlighting the importance of risk stratification and molecular profiling. The Seibert lab in San Diego addresses risk stratification in prostate cancer screening [1]. Niehusmann et al. focus on molecular profiling and inclusion of CNS-tumour patients in the national IMPRESS-Norway trial, and the paper presents work related to precision diagnostics and therapeutic implications in desmoplastic non-infantile ganglioglioma [2]. Moreover, Fjørtoft et al. in this special issue present a review focusing on the immune microenvironment upon breast cancer progression [3]. Increased understanding of disease mechanisms is important to continue to develop the precision cancer medicine field moving forward.

Session two focused on the need for innovative clinical study designs in the field of precision cancer medicine. Richard Schilsky from the University of Chicago presented the Targeted Agent and Profiling Utilization Registry (TAPUR) study [4], the planning of which inspired several of the European national initiatives, including the DRUP trial in the Netherlands. TAPUR is a pragmatic, multi-basket, non-randomized trial where targeted FDA (U.S. Food & Drug Administration) approved drugs are used outside indication. Results from TAPUR show that 34 cohorts have been completed [5]. Emile Voest from the DRUP study [6] highlighted how a network of DRUP-like clinical trials across Europe collaborate to share data and combine cohorts across trials, greatly enhancing the impact of the individual national initiatives. In this *Acta Oncologica* special issue, these large European consortia and their impact are described in more detail [7]. Furthermore, there is still a need for new innovative clinical trial designs as highlighted by Christophe Le Tourneau from the Institut Curie in Paris, and Voest also presented the novel DRUP ATTAC study design, offering combinatorial treatment in the presence of multiple molecular targets.

Session three addressed how precision cancer medicine challenges established models for reimbursement, and there is, thus, a need for policy invasion to facilitate implementation of precision oncology. Sahar B. van Waalwijk van Doorn-Khosrovani from the National Funder’s Committee for Evaluation of Specialised Medicines and Companion Diagnostics, CZ Health Insurance, The Netherlands explained how the risk-sharing reimbursement model in the DRUP and DAP (Drug Access Protocol) studies addresses the challenges when reimbursement decisions are made based on single-arm trials. The risk-sharing reimbursement models handle uncertainties regarding evidence and costs to maintain the sustainability of the healthcare system. Katarina Steen Carlsson from the Swedish Institute for Health Economics reflected on how existing Health Technology Assessment (HTA) models can be adapted to facilitate reimbursement decisions in precision cancer medicine. Moreover, Bettina Ryll from the Stockholm School of Economics Institute for Research described how a national multi-stakeholder ecosystem is necessary for precision cancer medicine implementation. She also highlighted how the European DRUP-like trial community is a self-organizing open innovation ecosystem interacting with national decision-makers, payers, HTA, commercial sector, and civil society [7]. Monika Frenzel from the French National Research Agency described the European funding programmes for personalised medicine. In particular, Frenzel presented the European Partnership for Personalised Medicine (EP PerMed) programme that was launched towards the end of 2023. This strategic platform will run for 10 years with an approximate budget of 330 million Euros.

Session four focused on scaling of precision medicine ecosystems. Technology scaling is a major challenge when broadening precision cancer medicine initiatives to a national level. Jesus Garcia-Foncillas from the Jiménez Diaz Foundation University Hospital in Madrid and Benedikt Westphalen from the Munich Comprehensive Cancer Center shared their experiences in the rapidly evolving precision cancer medicine landscape. Kadri Toome from Tartu University Hospital, presented the results from the Estonian initiative where the National Health Insurance Fund is financing tumour profiling at a national level. Estonia is currently in the process of establishing a DRUP-like clinical trial, EstOPreT [7].

The final session on the growing ecosystem of DRUP-like clinical trials and the European-wide initiatives PCM4EU and PRIME-ROSE [7], included updates from all ongoing DRUP-like clinical trials in Europe. Hans Gelderblom from Leiden University Medical Center presented the original DRUP trial [6]. The trial opened in 2016 and key elements to the DRUP success include MTBs, good research infrastructures, and involvement of payers and pharmaceutical companies. The latest update from the DRUP trial is presented by Mohammad et al. in this special edition [8]. Moreover, Gelderblom described how the first stage three expansion cohort using nivolumab for treatment of dMMR/MSI solid tumours met evaluation criteria, resulting in reimbursement of this treatment since July 2022 in the Netherlands. The second stage three cohort includes olaparib treatment of patients with BRCA mutated tumours. This cohort will include patients from several DRUP-like clinical trials. Åslaug Helland from Oslo University Hospital gave an update from the IMPRESS-Norway trial [9, 10]. The trial started accrual in April 2021 and has so far included 1167 patients in the molecular profiling phase. Of these, 31% had an actionable molecular alteration and a matching targeted drug eligible for inclusion in the treatment phase of the study [10]. According to Puco et al., 40% of the treated patients showed clinical benefit at 16 weeks [10]. IMPRESS-Norway has started recruitment of patients with biallelic BRCA1/2 inactivation to the stage three olaparib cohort, which is financed through public–private risk-sharing modelled after DRUP. Kristoffer Rohrberg from the Copenhagen University Hospital presented the ProTarget trial [11], which has been running for 3 years. ProTarget has so far evaluated 5000 genomic profiles and 185 patients have been treated in 112 cohorts. Katriina Jalkanen from the Helsinki University Hospital presented the FINPROVE trial at the conference, and an update is also published in this special issue [12]. The trial opened at the end of 2021, and so far, 310 patients have been evaluated and 85 patients have been offered treatment. Loic Verlingue from Centre Leon Berard in Lyon gave an overview of the multi-centric MOST trials MostPlus and MEGAMOST, with altogether 14 cohorts. MostPlus has so far treated 145 patients, and the latest update from the MOST trial family is presented in this precision cancer medicine edition [13]. The DETERMINE trial in the UK was presented by Matthew Krebs from the University of Manchester. This trial opened in November 2022 and is recruiting via existing national screening programmes. This Acta Oncology special edition presents two additional precision cancer medicine initiatives in Portugal [14] and Hungary [15], respectively. The recently opened Precision Oncology Platform (POP) trial is pioneering the implementation of a precision cancer medicine strategy in Portugal [14]. Toth et al. describe the application of comprehensive molecular genetic profiling in precision cancer medicine in Hungary [15], which is the first crucial infrastructure that needs to be in place for successful precision cancer medicine implementation. Altogether, there are several well-established national initiatives, and some of these are described in detail in this special issue. Kjetil Taskén from the Oslo University Hospital rounded off the NPCM conference with an overview of how the DRUP-like clinical trial communities collaborate through the EU-funded initiatives PCM4EU and PRIME-ROSE as also described in this issue [7].

## Conclusion

This first *ACTA Oncologica* Nordic Precision Cancer Medicine Symposium gathered renowned speakers from all over the world and facilitated increased international collaboration. The talks sparked good discussions and a vibrant and interactive environment. The next conference is planned for 2025. In this *Acta Oncologica* special issue, some of the addressed topics and relevant updates are described in more detail.
